# Does weather anomaly still affect the emerging stock market under the unexpected event? New evidence from the COVID-19 pandemic

**DOI:** 10.1016/j.heliyon.2023.e18665

**Published:** 2023-07-25

**Authors:** Nguyen Thi Hoa Hong, Pham Thi Mai Huong, Nguyen Yen Linh

**Affiliations:** a Financial Management-Statistics Analysis Department, Faculty of Business Administration, Foreign Trade University, Postal address: 91 Chua Lang Street, Dong Da District, Ha Noi, Viet Nam; b School of Economics and International Business, Foreign Trade University, Postal address: 91 Chua Lang Street, Dong Da District, Ha Noi, Viet Nam

**Keywords:** COVID-19, Cumulative abnormal return, Emerging stock market, Stock performance, Vietnam, Weather anomaly

## Abstract

This paper examines the impact of COVID-19 nationwide lockdown on the relationship between weather anomaly and the Vietnam stock market – a fast-growing emerging market. The paper employs event study methodology to compute the cumulative abnormal return of stocks during the pandemic, and the Holt-Winters Exponential Smoothing model to build the formula for weather anomaly for weather variables. In addition, a *t*-test is performed to examine the statistical significance of weather variables, as well as the impact that the lockdown order had on stock performance. Cross-sectional analysis by Ordinary Least Squares regression is also applied for estimating the relationship between weather and stock market performance. The finding shows that prior to the COVID-19 lockdown, all of the risk and return indicators, with the exception of idiosyncratic risk, are affected by temperature. After the lockdown order was withdrawn, temperature is only correlated with cumulative real returns and cumulative abnormal returns. Meanwhile, air pressure only appears to have an influence on cumulative abnormal returns after the lockdown, yet being the only meteorological factor that could impact the stock market during the lockdown. Generally, the larger the weather anomaly, the worse the returns and the higher the risks. The paper gives recommendations for listed companies and authorities to have better performance while engaging in and regulating the stock markets. Moreover, the results can be used as a reference for the investing community to incorporate meteorological factors into their analysis.

## 1 Introduction

There have been a number of studies demonstrating the various factors that could create fluctuations in the stock market. Namely, in terms of macroeconomic proxies, systematic economic news from industrial production and changes in the risk premium are found to price stock market returns [1], additionally, the negative effect of a sudden monetary tightening on the NASDAQ financial 100 index can last for around two months [2]. The stock market is also greatly influenced by the movement of regional stock markets, such as the stock markets of the G-20 nations [3], and the occurrence of terrorist attacks [4]. Beside these common economic factors, weather is an anomaly that has been given attention as its proxies are proven to produce a significant influence on various stock markets [[5], [6], [7], [8], [9]]. Saunders [10], for instance, studies the relationship between cloud cover and the three main listed stocks on the New York stock exchange market (DJIA, NYSE/AMEX value-weighted, and NYSE-AMEX equal-weighted) and finds that stock indices' returns are advanced by at least 6% on less cloudy days, which is a sign of better weather or sunny days. Whereas temperature is found to exert a significantly negative influence on Austria, Belgium and France stock market returns [7]. The evidence that weather changes can have an impact on investors' moods, which in turn reflects straightforward on the stock market, demonstrates that mood acts as a mechanism via which weather can affect the stock market. The link between meteorological factors and stock market indices is referred to as an anomaly since it departs from the efficient market hypothesis (EMH) as it was shown that changes in investors’ weather-induced mood had an impact on stock patterns [6].

Vietnam has long been one of the nation to be most vulnerable to climate threats, as determined by its level of exposure to and susceptibility to extreme weather occurrences. To be more specific, Vietnam is listed among the 11–20 nations most affected by extreme weather events from 2000 to 2019 in the Germanwatch Global Climate Risk Index 2021 report [11]. Taking the annual occurrence of strong storms, tropical depressions, below-13-degree Celsius days, heatwaves, extremely heavy rain days, and drought months as indicators for Vietnamese exposure to climate change and severe weather events, Tran [12] indicates that the exposure level in 2013 was very high, scoring 4.4 out of 5.0, and was high during the 2014–2017 period. It is evident that Vietnam is weather-sensitive, therefore, we raise the question of whether, aside from economic factors such as monetary policy, exchange rate appreciation, and GDP growth [13], the stock market is influenced by weather anomaly. Notably, COVID-19, one of the greatest pandemics to ever strike the world with over 700 million confirmed cases and more than 6 million fatalities as of June 2023, has recently spread throughout the world [14]. Surprisingly, Vietnam is one of the few nations that has successfully controlled the pandemic even from the beginning, despite its close proximity to China, limited healthcare system and resources, dense population, and especially with its over 18 million international visitors and 85 million domestic travelers recorded in 2019 [15]. To be more specific, according to the WHO Coronavirus Disease Situation Report 132 [16], when the total number of cases worldwide reached over 5.9 million and there were over 300.000 fatalities by May 31, 2020, Vietnam only had 328 cases overall, and there was even no death. At that time, there were over 1.7 million cases in the United States, whereas the site of the original pandemic, China, had 84.570 cases recorded. It can be seen that despite its high probability of being severely affected by COVID-19, Vietnam has successfully overcome the great pandemic owing to the nation's decisive strategy. In particular, on January 23, 2020, Viet Nam reported the first two confirmed cases of COVID-19. On the same day, the Prime Minister issued Official Telegram No. 121/CĐ-TTg which gives a summary of the current pandemic situation and orders competent authorities to coordinate and implement strict health-check controls at land, waterway border gates, and airports. All flights from and to Wuhan were also halted by the Civil Aviation Authority of Vietnam. On January 30, 2020, the Prime Minister continued to issue Decision No. 170/QĐ-TTg on the establishment of a National Steering Committee for COVID-19 Prevention and Control. It appears that the Vietnamese government recognized the severity of the pandemic and actively disseminated information to its citizens while implementing strict controls, starting a month before the World Health Organization (WHO) officially declared COVID-19 a pandemic on March 11, 2020. Following the pandemic emergency, the country continued to give strict directives. There were at least 110 laboratories across the country that can run the COVID-19 test in real time, with a daily sample capacity of 27,000 [17]. Additionally, in order to inform citizens about preventive or anticipatory actions, alert them to high-risk areas, and provide other services, the government also developed websites and mobile applications. The nationwide lockdown was implemented, in particular, on April 1, 2020, yet there was a noticeable rise during the final trading session of the month. In detail, the Hanoi Stock Exchange (HNX) index rose by 15.33% in comparison to the last March record [18]. According to Hartley et al. [19], Vietnam success can be attributed to six factors, which include command and control governance; extensive planning; fostering cooperative sentiment and solidarity; political readiness and communication; policy coordination; and adaptation. This could be instructive information that other countries might consider addressing the ongoing COVID-19 circumstance and other unexpected events. Therefore, Vietnam's success serves as a typical instance of how an emerging market with limited resources and high susceptibility can handle crises and the value of timely, deliberate action when confronted with unforeseen events.

The subject of the relationship between weather anomaly and stock markets has been considered in investigations for decades, however, the matter has received little attention in Vietnam, despite the country being highly sensitive to weather changes. Hence, we attempt to devote to the growing literature on the relationship between weather factors and the stock market by examining this nexus in the context of the Vietnam stock market. In addition, as opposed to prior research that focused on daily weather data, our investigation focused on weather anomaly, which describes differences between the actual weather and what is expected or forecasted to show how investors' trading outcomes will be impacted by weather-anomaly-induced mood. Moreover, the previous studies were mostly conducted over a certain period of time without taking the specific context of the socioeconomic environment into consideration. Especially, the globe has just gone through the unprecedented COVID-19 pandemic, which resulted in severe losses and significant change worldwide. The pandemic has led to lockdowns and other emergency measures, which have reduced people's exposure to weather changes. Given this situation, we are interested in exploring whether weather anomaly still affects investors who are working from home during the lockdown, then affecting the Vietnam stock market. Since the stock market in Vietnam has been experiencing an uptrend when the country has been under lockdown, our study may provide insight into whether weather is one of the explanatory elements that could influence the stock market. Following that, our study provides details on how weather anomaly impacts Vietnam, an emerging market that is particularly sensitive to weather. Collectively, the goal of our study is to examine this relationship for the three time periods of before, during and after the national lockdown on the Vietnam stock market.

Our study makes four contributions to the growing literature. Firstly, we fill the gap of previous studies, which are conducted under normal condition, by providing further evidence of the effect of weather anomaly on the emerging stock market under an unexpected event – COVID-19. Secondly, whereas the previous studies examined the weather impact on stock returns, volatility, and trading volume [7,10,20], our paper takes interest in that effect on cumulative real returns, cumulative abnormal returns, systematic risk, and idiosyncratic risk. Also, we employ the event study and Ordinary Least Squares (OLS) methods, which are appropriate for our cross-data and effectively capture changes of stock market under different COVID-19 periods in Vietnam. In addition, we build the formula of weather anomaly for weather variables using the Holt-Winters Exponential Smoothing model. Thirdly, we demonstrate how Vietnam stock performance varied over three time periods: before, during, and after the COVID-19 national lockdown. The finding shows that prior to the COVID-19 lockdown, all of the risk and return indicators, with the exception of idiosyncratic risk, are affected by temperature. After the lockdown order was withdrawn, temperature is only correlated with cumulative real returns and cumulative abnormal returns. Meanwhile, air pressure only appears to have an influence on cumulative abnormal returns after the lockdown, yet being the only meteorological factor that could impact the stock market during the lockdown. Generally, the results reveal that the larger the weather anomaly, the worse the returns and the higher the risks. Fourthly, based on our research, we recommend that investors should be aware of how weather anomaly can affect their decisions and avoid making rash decisions by developing long-term strategies and making informed decisions. Therefore, the governments should encourage a transparent and well-informed stock market. Additionally, when regulating the market, governments might take into account variables that may affect investors' behaviour, which include weather variables, in order to build appropriate regulations that gain the confidence of investors, encourage rational trading, and eventually increase market profitability.

The remainder of this paper is organized as follows. Section 2 reviews the literature and develops the research hypotheses. Section 3 presents the research design. The empirical results and discussion are reported in Section 4. Finally, Section 5 summarizes the major findings and provides some recommendations.

## 2 Literature review and hypotheses development

There have been many extensive studies conducted to examine the relationship between weather and the stock exchange market. Firstly inspired by Saunders [10], latter investigations continue to provide evidence of an existing correlation between meteorological variables and stock indices in various markets [[5], [6], [7], [8], [9]]. Cloudiness, for example, has a statistically significant impact on New York stock returns, according to Saunders [10]. In particular, the data collected on cloud cover and the three main listed stocks on the stock exchange market (DJIA, NYSE/AMEX value-weighted, and NYSE/AMEX equal-weighted) revealed that stock indices’ returns are found to advance by at least 6% on less cloudy days, which mean better weather or sunny days. While Saunders [10] used observations of the locations physically closest to Wall Street, Hirshleifer and Shumway [5] used simple city-by-city specifications with OLS regressions to investigate 26 leading stock exchanges across the world and also report a positive correlation between stock returns and sunny days. Sunshine seems to be the sole weather variable having an extremely significant influence on 26-country stock market returns in 1982–1997, as no linkage is found between rain, snow and returns. Cao and Wei [6] extended the scope of the study to nine stock indices from the United States, Britain, Germany, Canada, Sweden, Japan, Taiwan and Australia, which are all mature markets and geographically diversified, together with temperature, as it is well known for being one of the important weather variables. By implementing two types of tests - the bin test and regression analysis - to establish the association between temperature and stock returns, the results indicated that decreasing temperature leads to higher returns as low temperature is related to aggression in taking risk. Temperature has a negative impact across all of the countries studied, though to varying degrees. The influence of the daily New York temperature on the return as one standard deviation is consequently 0.029%, which is somewhat higher than one-half of the daily average return of 0.051%. Similar to how it is for Sweden, the temperature impact is roughly 1.5 times the daily average return and 4 times the daily average return for Taiwan.

The reason why the weather could affect the stock market is further explained by behavioural finance. Due to the evidence that stock patterns are affected by investors' weather-induced mood, the association between meteorological elements and stock market indices is referred to as an anomaly since it deviates from the Efficient Market Hypothesis (EMH) [6]. Specifically, behavioural finance's argument against traditional finance is that there is evidence that the stock market deviates from efficiency due to irrationality, which is partially brought about by investors' weather-induced moods. To be more specific, traditionalists all attempt to explain market movements by assuming that the market functions efficiently and is populated by consistently rational investors. According to the Efficient Market Hypothesis, the market remains efficient even if some investors are irrational and make mistakes when processing information given that they are uncorrelated, their mistakes cancel each other out [21]. Even when the asset prices deviate significantly from the fundamental values due to the mispricing now caused by a large number of investors, the mispricing will be corrected swiftly by the rational arbitrageurs as they find potential risk-free interest when there is an anomaly discovered in the fundamental value of securities [22]. In contrast, behavioural finance theorists prove the limitations of arbitrage as the arbitrageurs will bear the fundamental risk and unpredictable expectations of irrational traders that prevent rational traders from liquidating their position as selling a close substitute security short can not generate enough earnings if any news pulls the mispriced stock down further [23]. Moreover, even if there is a perfectly relative substitute, the arbitrage approach is no longer successful if the market functions properly and drives up the price of the substitute. Though rational economic agents are supposed to cancel any irrational fluctuations in asset prices that occur, it seems impractical for them to correct those mispricings as the demands of overreacting investors are barely detectable. Therefore, the market cannot remove all irrationalities. In addition, as traditional finance is based on the assumption of an efficient market and rational investors, it ignores the sentiment of human beings. Whereas, the concept of bounded rational investors is a fundamental pillar of behavioural finance theory.

According to behavioural finance theory, the limits of cognition and the influence of mood on decision-making are the stems of irrationality. The irrationality of investors is manifested in their propensity towards using heuristics and biases while making decision. In terms of cognitive biases, Tversky and Kahneman [24] demonstrated that representativeness, availability, adjustability and anchoring are three types of heuristics that people are more inclined to use in an effort to simplify the process of estimating probability or making predictions, and as a result, they tend to make biased decisions. Taking availability as an example, availability refers to the likelihood of people when accessing the probabilities of events by the ease of recalling the instances. Investors are prone to selecting attention-grabbing stocks when determining investment categories, and then they decide which stocks to purchase depending on their preference [25]. Moreover, the decision-making process is deviated due to the mood effect. Namely, Shu and Chang [26] illustrated that investors tend to have optimistic estimations of asset prices, leading to deviations from their intrinsic values and making mood-based decisions instead of drawing on underlying analysis. Interestingly, early findings by Isen and Patrick [27]revealed that the feelings-induced willingness to take risk differs between various kinds of risk. In greater detail, under hypothetical risk, positive state subjects are discovered to have more risk-taking behaviours than control groups, despite the fact that both subjects are judged to be more prudent in high-risk situations. When faced with a real risk, elated people wager more recklessly when the likelihood of losing is lower, but act more cautiously than control subjects when the risk is significantly higher. Recent studies have still continued to apply the relationship between mood and behaviour to other decisions such as mobile payment adoption [28], choice of products [29], judgment of sleep quality [30]. The reason why mood can exhibit influence on behaviours is that mood functions as an affective fundament for ongoing processing and behaviour [31]. In addition, Forgas [32] developed The Affect Infusion Model (AIM), which gives a comprehensive explanation of how affect (including moods) influences behaviour judgment. To be more specific, affect serves as a directly informative function under heuristic processing and exhibits an indirect priming effect influencing judgments when mood-congruent memories are activated. Additionally, it is found that historically, real-life occurrences like bubbles and crises were influenced by the irrationality of investors' psychological characteristics. Despite the macroeconomic elements that triggered the global financial crisis of 2008, greed, which came from the use of leverage that led to spiraling debts and involvement in derivatives, defined the severity of the crisis. Due to overconfidence and the expectation of a perpetual bull market and never-losing investments, consumers overlooked the crisis's warning signs and discounted potential risks [33]. Consequently, due to the fact that the market is not always efficient, and investors' decision-making process is likely to be affected by their cognitive limits as well as their mood, this results in the appearance of weather-induced mood effect on stock market.

The findings of subsequent studies support the idea that investors' mood appears to be the intermediary mechanism by which weather could affect the stock market. To be more specific, risk-taking or risk-averse behaviours are a result of weather-induced fluctuations in mood [34]. As adverse weather conditions increase negative feelings, investors are more likely to make a safer choice and are not interested in high payoffs. Contrarily, investors who are in an optimistic mood due to weather-related factors tend to be more risk-tolerant, thus undeterred by low expected returns. Mood seems to be an intermediary channel through which weather conditions exert an influence on investors' risk preferences, resulting in stock market indices [34,35]. Consequently, the stock market's outcomes reflect the risk-based decisions of investors. The nexus between mood and other financial decisions has also been investigated. For instance, optimistic institutional investors who expose themselves to decreasing cloudiness are less likely to perceive overpricing and purchase more stocks. In contrast, an increase in deseasonalized cloud cover leads to a 3% perceived overpricing rise [36]. When adding investment amount to represent risk level, positive mood investors, connected with decreased cloud cover, have a tendency to increase their investment in crowdfunding [37]. Nonetheless, the nexus is varied for groups of investors and seasons as the mature investors' decision-making process of investment is less influenced by the weather-induced mood. In terms of M&A performance, negative weather-induced mood states limit managerial overconfidence and arrogance of UK listed firms, which lowers overpaying behaviours and enhances acquiring performance [38]. As a result, it is possible that mood acts as a mediator between the weather and investors' behaviours, which will reflect on stock market outcomes. Consequently, weather exerts an indirect impact on the stock market via influencing the investors' mood.

As aforementioned, much attention has been paid to investigating whether weather has an impact on the stock market. By adopting a non-linear threshold model with the GJR- GARCH (1,1) process, Chang et al. [39] contributed to previous conclusions of linkage between weather variables and the stock exchange market by adding humidity besides cloud cover and temperature in Taiwan from July 1997 to October 2003, however, humidity does not strongly influence returns. For cloudiness and temperature, the negative correlation is still found, but the underperformance of stock returns is reported in both extremely hot and extremely cold conditions. Notably, based on the Spanish stock market's exceptional context, Pardo and Valor [40] attempted to find a possible relation between sunshine hours, humidity, and stock prices during two different trading systems, which were the floor and computerized environment, from 1981 to 2000. Nonetheless, no evidence of a relationship between sunshine durations, humidity levels, and the Madrid Stock Exchange Index's prices is found. Not only limited to study returns, Muhlack et al. [20] evaluated the association between cloud cover, air pressure, precipitation, humidity, temperature, and wind speed on returns, trading volume, and volatility of DAX, MDAX, SDAX, and TecDAX on the German stock market from 2003 to 2017. When air pressure rises, three indices—DAX, MDAX, and SDAX—but not TecDAX, are likely to increase. In accordance with the characteristics of Germany's temperature, the rising temperature, which means better weather, induces increasing returns of DAX and MDAX. This finding contradicts the previous negative relationship found between temperature and returns. When the dependent variable is volatility, both wind speed and humidity lower the volatility of SDAX and TecDAX, respectively. Contrarily, there is no connection at all between precipitation, air pressure, or temperature and the volatility of any analyzed indices. Hence, the effect of different weather variables on the German stock market seems not to be uniform.

Collectively, the results tend to differ depending on research design, individual investors' sensitivity to weather changes, weather factors existing in each stock market, the stock market's indices selected, the context of the studied stock exchange market, the perceptions of active investors of good and bad weather, and so forth. In addition, the stock markets examined in previous studies are primarily the leading markets. For instance, Shahzad [41] selected the Hong Kong Stock Exchange, one of the major global markets that was founded in 1891, to be one of the researched stock exchanges. Along with the availability of temperature data, Cao and Wei [6] also took the market's maturity and geographical differences into account before selecting nine stock indices from eight different markets, including the United States, Britain, Germany, Canada, Sweden, Japan, Taiwan and Australia. Additionally, in the study of Pardo and Valor [40], the authors selected the Madrid Stock Exchange as the best representative of the Spanish market because it ranked fourth in Europe and the seventh most active market globally in 2000. Whereas the Vietnamese stock market has newly developed, with the Ho Chi Minh City Stock Exchange (HOSE) opening its doors in 2000 and Ha Noi Stock Exchange following five years later, though the nation had been through a long time of preparation. Specifically, Viet Nam started to employ the equitization of state-owned enterprises in 1992, founded the State Securities Commission of Viet Nam in 1996 and issued Decree No.48/1998/NĐ-CP about securities and the stock exchange market in 1998. In the initial period of 2000–2005, there were only 41 listed stocks with their total capitalization accounting for approximately 1% of the nation's GDP [42,43]. Our study would therefore provide greater insight into how an emerging stock market in a country with its weather-sensitive characteristic would behave, particularly under the circumstances of lockdown.

In addition, as opposed to the previous studies, which used daily weather data, we utilize the term “weather anomaly,” which characterizes differences between the actual weather and what is anticipated or forecasted, to demonstrate how investors' trading decisions will be altered by weather-anomaly-induced mood. The stock market will eventually reflect changes in investor mood. In the following part, we state the following hypotheses based on our objective of examining the connection between weather anomaly and the Vietnam stock market for the three time periods of before, during, and after the nationwide lockdown on the Vietnam stock market.

### 2.1 Before and after the national lockdown

On March 11, 2020, the World Health Organization (WHO) declared COVID-19 a pandemic. Nearly all the nations’ stocks declined in response to the rising number of confirmed cases and deaths. In the March 2020 crash, the most impacted industries were crude petroleum and oil services, which recorded a 77% decrease in returns, real estate, which recorded a 72% decrease in returns, and hospitality and entertainment, which recorded a 70% decrease in returns, according to evidence from 1500 firms in the S&P1500 index [44]. The sector that is taking advantage of COVID-19 is healthcare and medical devices, which have seen an increase of 25.58% in returns, along with food and grocery distribution, software and technology, and natural gas. On January 23, 2020, Vietnam also reported the first two confirmed cases of COVID-19. The trading session at the end of March 2020 saw the VN-Index fall 28% from its last record of 2019, resulting in a loss of USD 37.4 billion [45]. The authors also gave evidence of the adverse impact of COVID-19 on Vietnam stock returns before the national lockdown was ordered. As neither non-pharmaceutical nor pharmaceutical measures had been released, the citizens suffered from severe uncertainty and fear. Such high sentiment fluctuations would create higher stock volatility.

Vietnam has long been one of the nations most vulnerable to climate threats, as determined by its level of exposure to and susceptibility to extreme weather occurrences. To be more specific, Vietnam is listed among the 11–20 nations most affected by extreme weather events between 2000 and 2019 in the Germanwatch Global Climate Risk Index 2021 report [11]. Additionally, it was determined that unfavourable weather, together with food shortages, a weak financial system, constrained growth in manufacturing and agriculture, and ineffective government governance, were the root causes of Vietnam's hyperinflation from 1986 to 1992 [12]. Collectively, it is apparent that the Vietnam economy is weather-sensitive. Hence, after the lockdown order is lifted and people resume their normal activities, we assume that it is likely that the weather anomaly effect will continue to have an impact. Therefore, we expect that the weather anomaly is likely to exert a profound influence on the stock market before and after the national lockdown, and then we propose our first hypothesis as below.

Hypothesis 1a Before and after the nationwide lockdown, weather anomaly exerts a negative effect on the Vietnam stock market.

Hypothesis 1b Before and after the nationwide lockdown, weather anomaly exerts a positive effect on the Vietnam stock market.

### 2.2 During the COVID-19 nationwide lockdown

Lockdowns and other emergency measures had been implemented in response to the COVID-19 pandemic in an attempt to slow its rapid spread, however, these measures dramatically reduced people's exposure to weather variations. According to the highest daily decline in people activity, the maximum lockdown is acknowledged as the most effective strategy for decreasing people's mobility [46]. Consider the UK as an example, on March 23, 2020, the prime minister issued an order for a national lockdown, instructing citizens to remain at home, forbid meetings of more than two people, halt non-essential commerce, and only allow citizens to purchase groceries and medications [47]. Furthermore, the COVID-19 home confinement increases the amount of time spent sitting each day by more than 28% and makes people physically inactive [48]. As a result, the COVID-19 lockdown drastically decreases human mobility worldwide, hence minimizing exposure to weather fluctuations. Thus, it is likely that weather has little effect on investors, barely altering the stock market.

Additionally, Deng et al. [49] examined the stock markets of 11 countries and 1 special administrative region with a view to measuring how the global stock markets responded to COVID-19 policy, including lockdown orders and interest rate cuts. The authors found that the stock markets of the countries that ordered lockdown responded favourably, performing better after the policy announcement because the policies lowered investors' fear and uncertainty and increased confidence. In Vietnam, the COVID-19 also brought the certain effects on other industries in Vietnam [50]. The national lockdown was imposed on April 1, 2020, however, the last trading session of the month saw a significant recovery. In detail, the Hanoi Stock Exchange (HNX) index rose by 15.33% in comparison to the last March record [18]. Nonetheless, the reason why the Vietnam stock market recovered is due to the attractiveness of the emerging stock market itself, the strict COVID-19 prevention policy earning investors' trust, and the easy access to the stock market to make money while working from home in order to offset income losses brought on by COVID-19. The weather does not appear to have a crucial impact. In German, the Frankfurt Stock Exchange also recovered even during the lockdown, despite experiencing a 40% decline before. The resilience incentive is shown to be the result of investors' great attention to the stock market being prompted by social isolation and their intent to purchase underpriced stocks [51]. In addition, the high returns of the stock are considerably correlated with a stable investor mood (indicated by momentum and liquidity). Hence, the evidence that investors’ mood can affect the stock market during the COVID-19 lockdown is consistent with the evidence of non-COVID-19 investigations [52,53]. However, weather anomaly may no longer be a factor that stimulates mood during the lockdown as the investors are no longer fully exposed to it. Therefore, we predict that the Vietnam stock market is less inclined to be influenced by weather anomaly changes during the nationwide lockdown. Accordingly, we would like to propose our second hypothesis as follows.

Hypothesis 2a During the COVID-19 lockdown, weather anomaly has no effect on the Vietnam stock market as investors are no longer fully exposed to weather changes.

Hypothesis 2b During the COVID-19 lockdown, weather anomaly has a profound effect on the Vietnam stock market as flexible hours associated with remote work increases investors' exposure to weather changes.

## 3 Research design

### 3.1 Data and sampling

Our initial research sample consists of all companies listed on Ho Chi Minh City Stock Exchange (HOSE) and Hanoi Stock Exchange (HNX) between 2018 and 2020. However, the dataset excludes all companies belonging to Finance - Banking sector due to their distinctive characteristic and mechanism, which results in a distinctive policy and regulations applied to this type of business by the Vietnamese State. Secondly, we eliminate companies that lack some data in the financial statements and annual reports. As the information is compulsory and irreplaceable in order to run our research model, the securities codes that were not traded during the period more than a month are removed. Then, 580 Vietnamese listed companies are included in our final sample.

Regarding weather variables, we collect weather data based on the location of each listed company's headquarter. Air pressure, humidity and wind speed are recorded every day. Particularly, temperature is measured 4 times per day at 1 a.m., 7 a.m., 1 p.m., 7 p.m., whereas rainfall is measured twice at 6 a.m. and 12 a.m. Some of the missing observations are supplemented with data from the neighboring province which has the same weather patterns. Finally, derived from 172 meteorological stations, our data covers 60 out of 63 provinces and cities in Vietnam from 2010 to 2020. The highest density of companies are still big cities with Ho Chi Minh, Hanoi (27.79%, 25.56%, respectively) and 3 others, Dong Nai, Hai Phong and Da Nang (more than 4%). The other companies will be scattered in the 55 remaining provinces.

### 3.2 Empirical models and research methods

In order to examine the relationship between weather anomalies and the movements of the Vietnamese stock market under the impact of the COVID-19 lockdown, our study employs four main methodologies which are event study analysis (ESM), seasonal Holt-Winters smoothing method, *t*-test and ordinary least squares (OLS) regression modeling.

#### 3.2.1 Event study methodology

The event study methodology (ESM) is a widely utilized analytical tool in finance that aims to assess the impact of an event on securities' performance. In this study, the method is used to estimate the cumulative abnormal return of the sample traded stocks. The Capital Asset Pricing Model (CAPM) is employed as a theoretical framework for the analysis.

In this study, the estimation window was set from December 1, 2018 to November 30, 2019, prior to the onset of the COVID-19 pandemic. We perform a CAPM-based analysis on the data obtained from the estimation window in order to estimate the alpha and beta specific to each stock. The parameters were then used to compute expected stock returns for the event windows. The expected returns play the role as a benchmark to determine how the actual return in each event window deviated from what was expected, measured as the abnormal return.

(1) R_i,t_ – Rf_t_ = i+ β_i_(Rm_t_ – Rf_t_) + _i,t_

Where R_i,t_ denotes the actual return on stock i at time t (the closing price), Rf_t_ represents the risk-free rate at time t (the Vietnamese one-year bond yield), Rm_t_ denotes the market return at time t (VN-Index), i,β_i_ are the parameters specific to stock i and i,t denotes the error term of stock i at time t.

**Fig. 1:**
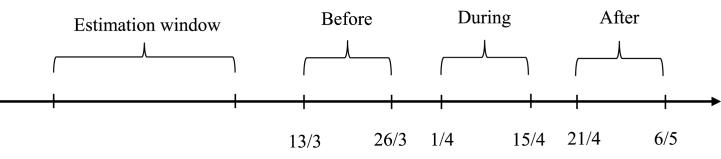
Timeline of research windows.

Our major empirical regressions estimating weather effects on the stock market are performed on three event windows, which are before, during and after the national lockdown as illustrated in Fig. 1 below. In the pre-lockdown period (Before), spanning from March 13 to March 26, COVID-19 had already spread globally and the Vietnamese people displayed a cautious attitude towards the pandemic, yet no lockdown order had been implemented by the government. The second event window (During) was established from April 1 to April 15 when the nationwide lockdown was in effect. During this period, people were primarily confined to their homes and avoided outdoor activities, with many opting to work from home, meaning exposure to weather conditions was limited. The post-lockdown event window (After) spanned from April 21 to May 6, marking a return to pre-pandemic normalcy as the restrictions imposed during the nationwide lockdown were lifted and businesses and daily operations resumed their normal functioning.

In our model, each window (Before, During, After) includes 10 trading days and those are 3 days apart each other in order to the effect of information leakage [54]. Estimation window lasts from 2018 to 2019 so as to increase the accuracy of the estimation.

Following Lakonishok and Shapiro [55]; Jiang and Zaman [56]; Rahman et al. [57], we measure the stock market performance by computing cumulative realized returns, cumulative abnormal returns, systematic risk and idiosyncratic risk for three above event windows.

Realized return is the return that is actually earned over a given time period. In our model, we calculate the realized return via the natural logarithm in Equation (2) below:

(2) $${RR}_{it}=\ln\frac{P_{it}}{P_{it-1}}$$

Where RR_it_ denotes the realized return of stock i at time t, P_it_ and P_it-1_ represent the prices of stock i at time t and t-1 respectively.

Therefore, the cumulative realized return over an event window is the sum of realized returns as in the following Equation (3):

(3) $${CRR}_{i}=\sum_{t1}^{t2}{RR}_{it}$$

Where CRR_i_ denotes the cumulative realized return of stock i, RR_it_ represents the realized return of stock i at time t and (t_1_, t_2_) is the event window.

Abnormal return of stock i at time t is the difference between realized return and an estimate of its expected (or normal) return in the absence of the event (COVID-19 in this situation). The formula is stated in the bellowing Equation (4):

(4) AR_it_ = RR_it_ - ER_it_

Where AR_i,t_ denotes the abnormal return of stock i at time t, RR_i,t_ is the actual return of stock i at time t and ER_i,t_ represents the expected return of stock i at time t.

The expected return is computed as Equation (5) bellows:

(5) ER_it_ = Rf_t_ + α_i_ + β_i_(Rm_t_ – Rf_t_)

Where ER_it_ is the expected return of stock i at time t; Rf_t_ represents the risk-free rate at time t; Rm_t_ denotes the market return at time t and α_i_ and β_i_ are the parameters of stock i estimated in Equation (1).

From Equation (4), cumulative abnormal returns (CAR) over an event window is the total abnormal return of stock:

(6) $${CAR}_{it}=\sum_{t1}^{t2}{AR}_{it}$$

Where CAR_it_ denotes the cumulative realized return of stock i, AR_it_ represents the abnormal return of stock i at time t and (t_1_, t_2_) is the event window.

In terms of risk, systematic risk refers to the risk that is inherent to the entire market, affecting the overall market, not limited to a particular stock nor industrial sector. Idiosyncratic risk refers to the inherent risk involved in an individual security or a very specific group of assets. Both systematic and idiosyncratic risks can be computed by running a linear regression on a stock's returns compared to the market using the capital asset pricing model (CAPM).

(7) R_it_ – Rf_t_ = α_i_ + β_i_(Rm_t_ – Rf_t_) + ϵ_it_

The systematic risk is measured by β_i_, while the idiosyncratic risk is the residual standard deviation.

#### 3.2.2 Seasonal Holt-Winters smoothing method

Weather factors variables are measured by Holt-Winters exponential smoothing method based on the research of Cao and Wei [6], Mirza et al. [58] in order to figure out the trend and seasonality in our data sample and implement forecasting models. Specifically in this study, we apply the additive seasonal Holt-Winters smoothing to the historical weather data spanning from 2010 to 2019, to compute the smoothing parameters and perform predictions for the study period in 2020. A forecast with additive Holt-Winters exponential smoothing can be expressed as Equation (8) below:

(8) F_t + k_ = L_t_ + (k × T_t_) + S_t + k-M_

In which L_t_ is the level estimate for time t, k is the number of forecasts into the future, T_t_ is the trend estimate at time t, S_t_ is the seasonal estimate at time t, and M is the number of seasons.

The estimation of smoothing parameters is performed separately for each province and city, since the meteorological features vary depending on geographic location, thus the seasonality and trend patterns are different among regions.

Weather anomaly is then computed as the difference between actual weather data and what is forecast by the Holt-Winters exponential method in the following Equation (9).

(9) Anom_t_ = |actual weather_t_ - forecast weather_t_|

Where Anom_t_ denotes the weather anomaly at time t, actual weather_t_ is the actual weather at time t and forecast weather_t_ is the forecast weather at time t.

The cumulative weather anomaly is computed as the sum of all weather anomalies in the study event window:

(10) $${CAnom}_{t}=\sum_{i=1}^{t}{Anom}_{i}$$

Where CAnom_t_ represent for the cumulative anomaly from time 1 to t and Anom_i_ is the weather anomaly at a specific time.

#### 3.2.3 Ordinary least squares (OLS) regression method

The ordinary least squares (OLS) estimation method is utilized to assess the relationship between weather anomalies and the stock market performance before, during and after the COVID-19 nationwide lockdown in Vietnam.

Specifically, our research computed the cumulative weather anomaly for each city over a specific study period, and estimate its effect on the financial performance of firms that are headquartered in these cities. We determined that OLS regression is the most appropriate method for analyzing our cross-sectional dataset. Dynamic models such as DOLS, FMOLS, PMG, MG, ARDL, and non-ARDL are commonly used for time series data, where the variables are observed over time, and may exhibit non-stationary behaviour, unit roots, or cointegration. Therefore, these models are not well-suited for our cross-sectional data. Similarly, GARCH models are typically used to model time-varying volatility or heteroskedasticity in financial data, and may not be necessary for our research question. Indeed, the use of OLS method to address similar research questions has been witnessed in a number of prior research studies [[59], [60], [61]].

The impact of weather anomalies on stock performance in the pre-, during, and post-lockdown period is estimated by a single regression model, but with different datasets from the three studied event windows. The dependent variables in the models are cumulative real return (CRR), cumulative abnormal return (CAR), systemic risk (Systemic_Risk), and idiosyncratic risk (Idiosyncratic_Risk), collectively referred to as the performance variables.

(11) $${Performance}_{i}=\beta_{0}+\beta_{1}{RAIN}_{i}+\beta_{2}{TEMP}_{i}+\beta_{3}{HUMID}_{i}+\beta_{4}{AIR}_{i}+\beta_{5}{WIND}_{i}+\beta_{6}{Longdebt}_{i}+\beta_{7}{Shortdebt}_{i}+\beta_{8}{Cash}_{i}+\beta_{9}{Profit}_{i}+\beta_{10}{TobinQ}_{i}+\beta_{11}{Size}_{i}+\beta_{12}{ROE}_{i}+\varepsilon_{i}$$

Where subscript i and t indicate the firm and year, respectively. All variables are described in Table 1 below.

**Table 1:** Definitions of the variables.

Variables	Explanation	Calculation	Expected sign	
			Performance	Risk
**Dependent variables (Performance)**				
CRR	Cumulative realized returns	Calculated in Eq. (3)		
CAR	Cumulative abnormal return	Calculated in Eq. (6)		
Systematic_Risk	Systematic risk	Calculated in Eq. (7)		
Idiosyncratic_Risk	Idiosyncratic risk	Calculated in Eq. (7)		
**Independent variables**				
RAIN	Rainfall	Calculated in Eq. (10)	–	+
TEMP	Temperature	Calculated in Eq. (10)	–	+
HUMID	Humidity	Calculated in Eq. (10)	–	+
AIR	Air pressure	Calculated in Eq. (10)	–	+
WIND	Wind Speed	Calculated in Eq. (10)	–	+
**Firm-level controlling variables**				
Shortdebt	Short-term debt	Natural logarithm of short term debt	−/+	−/+
Longdebt	Long-term debt	Natural logarithm of long term debt	−/+	−/+
Cash	Cash	Natural logarithm of cash	−/+	−/+
Profit	Net Profit Margin	Net Profit/Total Sales	−/+	−/+
TobinQ	Tobin's Q	Market value of a company/Asset replacement cost	−/+	−/+
ROE	Return on equity	Net profit/Total Shareholder's equity	−/+	−/+
Size	Firm size	Natural logarithm of the total asset	−/+	−/+

Notes: Table 1 presents the detailed calculations for each variable identified in our models as discussed in the (Empirical Models) section above.

## 4 Results and discussion

### 4.1 Overview of weather anomalies and stock performance

**Fig. 2:**
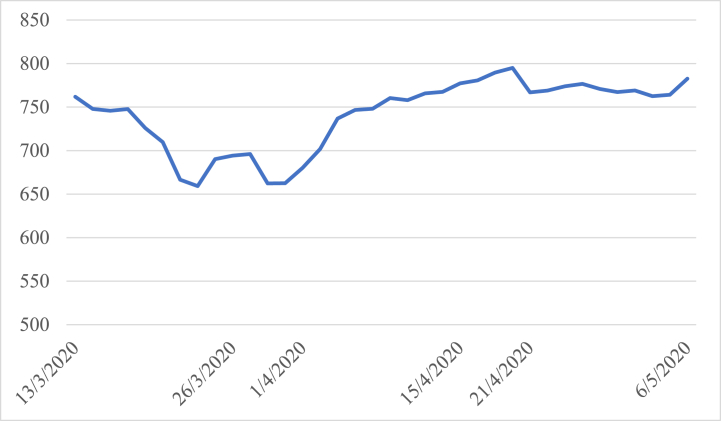
VN-Index performance during the study period.

As can be seen from Fig. 2, the Vietnamese stock market experienced a period of high volatility and instability before the national lockdown was implemented. The index dropped significantly from 761.78 points on March 13 to 694.21 points on March 26, due to the uncertainty surrounding the pandemic and its impact on the global economy. During the 10 trading days of the lockdown, the market began to recover gradually as a result of the government's response to the pandemic with proactive measures and remediations, with the VN-Index rebounding from as low as 680.23 points on April 1, to 777.22 points on April 15, the last day of the lockdown. However, in the 10 trading days preceding the lockdown, the market flattened, with the index fluctuating within a narrow range between 766.84 points and 782.59 points. This may be attributed to investors' cautiousness towards the uncertain economic outlook, as well as the market's adjustment to the new normal caused by the pandemic.

Prior to estimating the impact of weather on stock performance, one-sample *t*-test was conducted in order to examine the statistical significance of weather anomalies for the whole study period. The results, presented in Table 2, reveal the abnormality in weather factors is significantly different from zero, which suggests the actual weather deviates fairly from what was forecast.

**Table 2:** T-test results of weather anomalies.

Variable	Mean	Observation	S.E	S.D	95% Confidence interval	
Rainfall anomaly	22.325	1749	0.342	14.309	21.654	22.996
Temperature anomaly	23.753	1749	0.475	19.875	22.821	24.686
Humid anomaly	64.999	1749	0.943	39.447	63.149	66.849
Air pressure anomaly	32.937	1749	1.750	73.173	29.505	36.368
Wind speed anomaly	7.606	1749	0.118	4.921	7.375	7.837

Note: Table 2 presents one sample *t*-test results of weather anomalies for pre, during and after the COVID-19 national lockdown in Vietnam. The definitions of these variables are provided in Table 1.

In this study, we perform a paired-sample *t*-test in order to examine the influence of COVID-19 lockdown on the Vietnamese stock market. The test results are shown in Table 3 as follow.

**Table 3:** The difference between before and during, during and after.

	Before		During		After		Difference (Before - During)	Difference (During - After)
	Mean	S.D	Mean	S.D	Mean	S.D	t-value	t-value
CRR	−0.133	0.005	−0.002	0.005	−0.021	0.004	0.131***	−0.020***
CAR	−0.023	0.006	0.033	0.007	0.033	0.005	0.056***	0.000
Systematic risk	0.353	0.021	0.352	0.035	0.116	0.036	−0.001	−0.236***
Idiosyncratic risk	0.030	0.001	0.028	0.001	0.026	0.001	−0.002***	−0.002**

Note: Table 3 presents paired *t*-test results to examine the influence of COVID-19 lockdown on the Vietnamese stock market. The definitions of these variables are provided in Table 1.

In comparison to the pre-lockdown period, performance indicators during the restriction time experienced a significant increase while there was a decline in the idiosyncratic risk. This suggests the lockdown order alleviates risks and facilitates better market performance, as the government's response relieved investors from uncertainty and enhanced their confidence [49].

The results indicate a fall in the cumulative realized return (CRR) in the post-lockdown period, yet no significant difference is detected in the cumulative abnormal return (CAR). The systematic and idiosyncratic risks also experience a fair reduction. Collectively, it can be inferred that the policy imposed might have a favourable impact on the market, however, the influence mitigated over the time.

### 4.2 The impact of weather anomaly on the Vietnamese stock market before and after the lockdown

Table 4 presents OLS results for the effects of weather anomaly on the Vietnamese stock market before and after the COVID-19 national lockdown. During the pre-lockdown period, temperature anomaly had a negative impact on the stock return at the 95% level of confidence. As it is implied from the results, a difference of 1 °C from expected temperature was equivalent to 0.11- and 0.19-percent decline in cumulative realized return (CRR) and cumulative abnormal return (CAR), respectively. The results also indicate a positive correlation between anomaly in atmospheric temperature and systematic risk. Despite an interruption in the effect of temperature during the time when lockdown order was imposed, this weather factor continued to exert an adverse impact on the stock market returns after restriction measures were lifted on April 16, 2020. A similar relationship was detected between atmospheric pressure anomaly and the Vietnamese stock market. At 5% level of significance, higher anomaly in air pressure resulted in lower cumulative abnormal returns for both the pre- and post-lockdown period. However, there exists no correlation between air pressure and different risk indicators.

**Table 4:** The effect of weather anomaly on the stock market before and after the lockdown.

	CRR	CAR	Idiosyncratic risk	Systematic risk
**Before**				
Rainfall anomaly	0.0009 (0.96)	0.0017 (1.56)	0.0001 (0.49)	−0.0001 (−0.03)
Temperature anomaly	−0.0011** (−1.97)	−0.0019*** (−3.18)	0.0001 (1.22)	0.0043** (2.15)
Humid anomaly	−0.0001 (−0.24)	0.0004 (1.13)	0.0000 (0.22)	0.0019 (1.56)
Air pressure anomaly	−0.0001 (−0.44)	−0.0007*** (−2.94)	−0.0000 (−0.50)	−0.0011 (−1.34)
Wind speed anomaly	−0.0007 (−0.41)	0.0003 (0.16)	0.0004* (1.88)	0.0065 (1.12)
ROE	0.0221 (0.41)	−0.0882 (−1.49)	0.0072 (1.02)	0.2255 (1.17)
TobinQ	0.0153** (2.54)	0.0425*** (6.45)	−0.0029*** (−3.65)	0.0029 (0.14)
Shortdebt	0.0000 (0.66)	0.0000 (0.89)	−0.0000 (−1.57)	0.0000 (0.18)
Longdebt	0.0000 (0.01)	−0.0000 (−0.31)	0.0000 (1.03)	−0.0000*** (−3.65)
Cash	−0.0000 (−0.58)	−0.0000 (−0.36)	0.0000 (0.92)	0.0001 (1.79)
Profit	−0.0000 (−0.55)	0.0000 (−0.46)	0.0000 (0.31)	0.0000 (0.11)
Size	−0.0167*** (−3.73)	−0.0092* (−1.87)	0.0005 (0.85)	0.0958*** (5.97)
Constant	0.3192** (2.57)	0.1579 (1.16)	0.0123 (0.75)	−2.4585**** (−5.54)
Observation	574	572	574	574
R-squared	0.0599	0.1191	0.0405	0.1282
**After**				
Rainfall anomaly	−0.0007 (−1.46)	−0.0006 (−1.00)	−0.0000 (−0.46)	−0.0048 (−1.21)
Temperature anomaly	−0.0007** (−2.11)	−0.0008** (−1.98)	−0.0001 (−1.03)	0.0013 (0.47)
Humid anomaly	0.0000 (0.03)	−0.0001 (−0.59)	0.0000 (0.68)	0.0005 (0.44)
Air pressure anomaly	0.0000 (0.82)	−0.0002*** (−3.00)	−0.0000 (−0.33)	0.0001 (0.12)
Wind speed anomaly	0.0003 (0.28)	0.0004 (0.29)	0.0001 (0.80)	0.0008 (0.09)
ROE	0.1828*** (4.63)	0.2276*** (4.63)	0.0042 (0.62)	−0.2567 (−0.74)
TobinQ	−0.0023 (−0.62)	−0.0291*** (−6.24)	−0.0017*** (−2.65)	0.0321 (0.98)
Shortdebt	−0.0000 (−0.82)	−0.0000 (−0.56)	0.0000 (−0.60)	0.0000 (0.20)
Longdebt	0.0000 (0.89)	0.0000 (0.90)	0.0000 (0.03)	0.0000 (0.34)
Cash	0.0000 (0.62)	−0.0000 (−0.16)	−0.0000 (−0.25)	0.0001* (1.84)
Profit	−0.0000 (−0.17)	0.0000 (0.28)	−0.0000 (−0.04)	0.0000 (0.31)
Size	0.0115*** (3.41)	0.0037 (0.88)	0.0004 (0.71)	0.0404 (1.36)
Constant	−0.3150*** (−3.43)	−0.0046 (−0.04)	0.0165 (1.04)	−0.9684 (−1.21)
Observation	569	569	569	569
R-squared	0.0889	0.1114	0.0214	0.0506

Notes: This table presents Pooled OLS regression results for the effects of weather anomaly on the Vietnamese stock market before and after the lockdown of Eq. (11): ${Performance}_{i}=\beta_{0}+\beta_{1}{RAIN}_{i}+\beta_{2}{TEMP}_{i}+\beta_{3}{HUMID}_{i}+\beta_{4}{AIR}_{i}+\beta_{5}{WIND}_{i}+\beta_{6}{Longdebt}_{i}+\beta_{7}{Shortdebt}_{i}+\beta_{8}{Cash}_{i}+\beta_{9}{Profit}_{i}+\beta_{10}{TobinQ}_{i}+\beta_{11}{Size}_{i}+\beta_{12}{ROE}_{i}+\varepsilon_{i}$. The definitions for the variables are provided previously. *, **, and *** indicate statistical significance at 10%, 5%, and 1%, respectively. *4.3. The impact of weather anomaly on the Vietnamese stock market during the lockdown period*.

Regarding the three remaining weather factors, namely rainfall, humidity and wind speed, their impact on the market was far from being significant. The results are consistent with previous research by Goetzmann et al. [36], Giang et al. [62]. This is consistent with our initial anticipation, based on a number of research studies which provided evidence of an existing correlation between meteorological variables and stock indices [[5], [6], [7], [8], [9]]. This relationship can be comprehensively explained through the mechanism of weather-induced mood changes, which in turn influence decision making, trading behaviours and ultimately, market performance.

While previous investigations shared the same conclusion that the affective state of individual investors is subject to weather fluctuations [63,64], the results were conflicting as to in which direction weather conditions affect mood. It was indicated by Cao and Wei [6] that high temperature, as a weather factor, can induce apathy as well as hysteria and vice versa. However, it is widely accepted that the more the weather deviates from normal conditions, the higher likelihood that investors experience discomfort and negative feelings. As a result, they tend to make a safer choice and are not interested in high payoffs, which consequently leads to a decline in returns [34]. Studies by Yuen and Lee [65] and Kliger and Levy [66] also found evidence supporting the conclusion that the more elated the investors' mood, the more risk-averse they become. Hence, it is undeniable that mood seems to be an intermediary channel through which weather conditions influence financial investors’ risk preferences and gains from investment [34,35]. Given that the demand for equities is ultimately driven by investors, and their actions can markedly affect the market, increases in weather anomaly indirectly exert a harmful impact on stock performance.

To conclude, our findings about the negative relationship between temperature, atmospheric pressure anomalies and market performance are in line with preceding theories in meteorology, human behaviour and finance study. Given that, it is impossible to completely reject Hypothesis 1a as the impact of temperature and air pressure on the stock performance remained evident throughout the study period. Besides, the study also contributes to reaffirming behavioural finance, which has demonstrated that investors' behaviours can deviate from rationality due to cognitive limitations and affective states.

Our study also investigates the effects of weather anomaly on the Vietnamese stock market during lockdown in order to more comprehensively assess the impact of Covid-19 on the relationship between the weather and the stock market. The results from OLS regression models are presented in Table 5. During the lockdown, air pressure is the only factor to have an impact on the stock market. As is implied by the results, higher anomaly in atmospheric pressure caused a decrease in the cumulative abnormal return, or in other words, negatively affecting the stock market. Temperature anomaly which exerted adverse impact on both the pre- and post-lockdown markets, failed to have any influence during the time restriction measures were carried out. Additionally, the anomaly in the remaining meteorological factors, namely precipitation, humidity and wind speed, showed no statistical significance. Therefore, while Hypothesis 2b has not been entirely refuted, it is evident that our research results are more inclined to support Hypothesis 2a compared to Hypothesis 2b.

**Table 5:** The effect of weather anomaly on the stock market during the lockdown.

	CRR	CAR	Idiosyncratic risk	Systematic risk
Rainfall anomaly	0.0003 (1.11)	0.0003 (0.65)	0.0000 (0.01)	0.0004 (0.17)
Temperature anomaly	−0.0000 (−0.05)	0.0004 (0.79)	−0.0000 (−0.38)	0.0030 (1.31)
Humid anomaly	0.0002 (0.89)	−0.0003 (−1.12)	−0.0000 (−0.29)	−0.0003 (−0.20)
Air pressure anomaly	−0.0000 (−0.51)	−0.0004*** (−5.3)	−0.0000 (−0.32)	−0.0003 (−0.80)
Wind speed anomaly	0.0012 (0.49)	0.0027 (0.88)	0.0000 (0.02)	0.0291 (1.86)
ROE	0.1770*** (3.55)	0.2830*** (4.39)	0.0008 (0.11)	1.0208*** (3.08)
TobinQ	−0.0008 (−0.17)	−0.0465*** (−7.57)	−0.0023*** (−3.3)	−0.0292 (−0.92)
Shortdebt	−0.0000 (−0.21)	−0.0000 (−0.03)	−0.0000 (−0.55)	0.0000 (0.68)
Longdebt	−0.0000 (−0.33)	−0.0000 (−0.10)	−0.0000 (−0.41)	−0.0000 (−1.55)
Cash	0.0000 (1.52)	0.0000 (0.40)	0.0000 (0.15)	0.0000 (0.76)
Profit	−0.0000 (−1.75)	−0.0000 (−0.97)	−0.0000 (−0.95)	−0.0002 (−1.26)
Size	0.0274*** (6.46)	0.01389** (2.53)	0.0009 (1.47)	0.1154*** (4.10)
Constant	−0.7949*** (−6.74)	−0.3224** (−2.12)	0.0064 (0.37)	−3.1103*** (−3.98)
Observation	569	569	569	569
R-squared	0.1413	0.1574	0.0326	0.0778

Notes: This table presents Pooled OLS regression results for the effects of weather anomaly on the Vietnamese stock market during the lockdown of Eq. (11): ${Performance}_{i}=\beta_{0}+\beta_{1}{RAIN}_{i}+\beta_{2}{TEMP}_{i}+\beta_{3}{HUMID}_{i}+\beta_{4}{AIR}_{i}+\beta_{5}{WIND}_{i}+\beta_{6}{Longdebt}_{i}+\beta_{7}{Shortdebt}_{i}+\beta_{8}{Cash}_{i}+\beta_{9}{Profit}_{i}+\beta_{10}{TobinQ}_{i}+\beta_{11}{Size}_{i}+\beta_{12}{ROE}_{i}+\varepsilon_{i}$. The definitions for the variables are provided previously. *, **, and *** indicate statistical significance at 10%, 5%, and 1%, respectively.

In explaining for the discontinued impact of temperature specifically, it is worth considering that according to the definition of the National Oceanic and Atmospheric Administration (NOAA), weather refers to the atmospheric conditions at a specific location over a short period of time that people encounter when they go outdoors. When lockdown order was imposed nationwide, however, investors were, most of the time, confined insides, which means natural atmospheric temperature had fewer opportunity to exert an impact on investors. Previous studies by Sadowski et al. [46] and Ammar et al. [48] shared a conclusion that the COVID-19 lockdown drastically decreased human mobility worldwide, hence minimizing their exposure to weather fluctuations. Yan et al. [67] provided evidence of an increase in the use of air conditioners when people stay at home, meaning that natural atmospheric temperature had fewer opportunity to exert an impact on investors amid lockdown as the temperature was fixed by their subjective demands.

The impact of atmospheric pressure anomaly, however, was still prevalent even when the restriction order was imposed and outdoor activities were stringently restricted. From a broader view, it can be concluded that atmospheric pressure was the only weather phenomenon to have an influence on the cumulative abnormal return over the three periods under examination, regardless of the lockdown. This is reasonable given the fact that people still experience changes in atmospheric pressure even when being confined to indoor spaces, while other meteorological conditions can hardly exert any impact on them once outdoor activities are limited [68]. Furthermore, air pressure is shown to have an indirect correlation with investors’ mood [69], whereas Bassi et al. [34] proved that mood affects trading behaviours. Collectively, the preceding literature provides comprehensive explanation for the persistent impact of air pressure effect on the stock market, even during the nationwide lockdown.

In contrast, the insignificance of precipitation, humidity and wind speed was consistent throughout the pre-, lockdown and post-lockdown periods. This finding is reasonable, since several studies found no correlation between humidity, rainfall and market returns [39,70]. Additionally, the majority of firms listed on the Vietnamese stock exchanges are categorized into sectors such as technology, basic materials and consumer staples, which include no weather-sensitive industries.

### 4.3 Robustness test

In order to confirm the validity of the main findings, we perform a robustness test on the same data sample, yet employing the standard deviation of meteorological elements over the study period as a different proxy for changes in weather conditions.

The robustness check results presented in Table 6 are generally consistent with the major outcomes of our research in terms of coefficients and significance levels. The results provide additional evidence to support the conclusion that weather variability can affect the stock market, but this effect alleviated during the time restriction order was implemented. Specifically, while temperature volatility had a negative impact on stock performance both before and after the lockdown, atmospheric pressure is the only effective factor during the period. This can be explained by the fact that investors were still exposed to the influence of air pressure even when they are confined to indoor space amid the restriction time.

**Table 6:** Robustness test results of before, during and after the lockdown.

	CRR	CAR	Idiosyncratic risk	Systematic risk
**Before**				
St Rainfall	0.0047 (0.61)	0.0119 (1.40)	−0.0015 (−1.51)	−0.0015 (−1.51)
St Temperature	−0.0262** (−2.05)	−0.0194 (−1.36)	0.0028* (1.67)	0.0028* (1.67)
St Humidity	−0.0024 (−0.30)	−0.0057 (−0.64)	0.0004 (0.38)	0.0004 (0.38)
St Air pressure	0.0298 (1.55)	0.0027 (0.13)	−0.0016 (−0.62)	−0.0016 (−0.62)
St Wind speed	−0.0452 (−1.11)	−0.0377 (−0.83)	0.0043 (0.80)	0.0043 (0.80)
ROE	0.0192 (0.35)	−0.0940 (−1.56)	0.0087 (1.21)	0.0087 (1.21)
TobinQ	0.0154** (2.56)	0.0425*** (6.37)	−0.0029*** (−3.66)	−0.0029*** (−3.66)
Shortdebt	0.0000 (0.73)	0.0000 (0.93)	−0.0000* (−1.71)	−0.0000* (−1.71)
Longdebt	0.0000 (0.00)	−0.0000 (−0.35)	0.0000 (1.08)	0.0000 (1.08)
Cash	−0.0000 (−0.57)	−0.0000 (−0.25)	0.0000 (0.92)	0.0000 (0.92)
Profit	−0.0000 (−0.54)	−0.0000 (−0.44)	0.0000 (0.35)	0.0000 (0.35)
Size	−0.0170*** (−3.82)	−0.0100** (−2.02)	0.0006 (0.97)	0.0006 (0.97)
Constant	0.3292*** (2.65)	0.2677* (1.94)	0.0122 (0.74)	0.0122 (0.74)
Observation	574	572	574	574
R-squared	0.0676	0.0960	0.0434	0.1368
**During**				
St Rainfall	0.0019 (1.15)	0.0014 (0.63)	0.0000 (0.07)	0.0045 (0.40)
St Temperature	0.0227 (1.47)	0.0084 (0.41)	0.0036 (1.57)	0.1609 (1.56)
St Humidity	0.0044 (0.96)	−0.0044 (−0.72)	−0.0003 (−0.50)	0.0298 (0.97)
St Air pressure	−0.0484** (−2.51)	−0.0150 (−0.58)	−0.0053* (−1.86)	−0.1079 (−0.84)
St Wind speed	0.0666 (1.52)	0.0166 (0.28)	0.0099 (1.54)	0.3554 (1.22)
ROE	0.1709*** (3.43)	0.2714*** (4.10)	0.0010 (0.14)	1.0236*** (3.08)
TobinQ	−0.0009 (−0.19)	−0.0455*** (−7.23)	−0.0023*** (−3.26)	−0.0289 (−0.92)
Shortdebt	−0.0000 (−0.28)	−0.0000 (−0.10)	−0.0000 (−0.70)	0.0000 (0.59)
Longdebt	−0.0000 (−0.23)	−0.0000 (−0.02)	−0.0000 (−0.29)	−0.0000 (−1.45)
Cash	0.0000 (1.54)	0.0000 (0.37)	0.0000 (0.18)	0.0000 (0.78)
Profit	−0.0000* (−1.81)	−0.0000 (−0.91)	−0.0000 (−1.05)	−0.0002 (−1.33)
Size	0.0259*** (7.09)	0.0142** (2.51)	0.0009 (1.41)	0.1141*** (4.03)
Constant	−0.7546*** (−6.15)	−0.3190* (−1.96)	0.0044 (0.24)	−3.2161*** (−3.94)
Observation	569	567	569	569
R-squared	0.1474	0.1132	0.0401	0.0781
**After**				
St Rainfall	−0.0035 (−0.89)	0.0056 (1.12)	0.0011 (1.57)	−0.0019 (−0.06)
St Temperature	−0.0152** (−2.16)	−0.0119 (−1.35)	0.0004 (0.35)	0.0258 (0.42)
St Humidity	0.0089 (1.34)	0.0009 (0.10)	0.0016 (1.45)	−0.0519 (−0.89)
St Air pressure	−0.0026 (−0.59)	−0.0032 (−0.59)	−0.0008 (−1.05)	0.0508 (1.33)
St Wind speed	−0.0039 (−0.15)	0.0135 (0.42)	0.0042 (0.95)	0.1845 (0.83)
ROE	0.1841*** (4.65)	0.2221*** (4.46)	0.0032 (0.48)	−0.2475 (−0.71)
TobinQ	−0.0025 (−0.65)	−0.0286*** (−6.07)	−0.0016** (−2.54)	0.0316 (0.96)
Shortdebt	−0.0000 (−0.84)	−0.0000 (−0.56)	−0.0000 (−0.65)	0.0000 (0.19)
Longdebt	0.0000 (0.93)	0.0000 (0.93)	0.0000 (0.13)	0.0000 (0.32)
Cash	0.0000 (0.66)	−0.0000 (−0.15)	−0.0000 (−0.29)	0.0001* (1.87)
Profit	−0.0000 (−0.14)	0.0000 (0.32)	−0.0000 (−0.02)	0.0001 (0.35)
Size	0.0110*** (3.25)	0.0033 (0.78)	0.0003 (0.44)	0.0422 (1.42)
Constant	−0.3340*** (−3.51)	−0.0384 (−0.32)	0.0120 (0.74)	−1.1444 (−1.37)
Observation	569	567	569	569
R-squared	0.0919	0.0944	0.0346	0.0489

Notes: This table presents Robustness test results for the effects of weather anomaly on the Vietnamese stock market before, during and after the lockdown of Eq. (11): ${Performance}_{i}=\beta_{0}+\beta_{1}{RAIN}_{i}+\beta_{2}{TEMP}_{i}+\beta_{3}{HUMID}_{i}+\beta_{4}{AIR}_{i}+\beta_{5}{WIND}_{i}+\beta_{6}{Longdebt}_{i}+\beta_{7}{Shortdebt}_{i}+\beta_{8}{Cash}_{i}+\beta_{9}{Profit}_{i}+\beta_{10}{TobinQ}_{i}+\beta_{11}{Size}_{i}+\beta_{12}{ROE}_{i}+\varepsilon_{i}$. The definitions for the variables are provided previously. *, **, and *** indicate statistical significance at 10%, 5%, and 1%, respectively.

## 5 Conclusion and recommendations

Our paper aims to determine the correlation between the weather and the Vietnamese stock market under the COVID-19 pandemic. There are four main methods that are utilized, namely event study method, OLS method, *t*-test and seasonal Holt-Winters smoothing method. The results indicate that generally, the weather anomalies do have an impact on the stock market meanwhile the effect was limited during the lockdown with air pressure being the only influential factor. The overall result is in line with earlier research [[5], [6], [7], [8], [9], [10]]. Specifically, all performance measures increased significantly during the nationwide lockdown, with the exception of idiosyncratic risk. The outcome confirmed the effectiveness of the government's COVID-19 response strategy in earning the confidence and trust of investors [45,49]. However, all the effects only came from air pressure. This is reasonable considering that the COVID-19 lockdown greatly limited human mobility worldwide, reduced their exposure to weather changes [46,48], and increased the usage of air conditioners when people were at home [67]. For the both period of before and during lockdown, our study revealed a negative relationship between temperature, atmospheric pressure anomalies and market performance, which is consistent with the previous studies of the relationship between weather-induced mood and investors' behaviours. Particularly, Bassi et al. [34] indicated that the more the weather deviates from normal conditions, the greater the likelihood that investors will feel uncomfortable and negatively affected. Consequently, people frequently choose the safer option and are not motivated by large payoffs, which causes returns to drop.

However, unpredictable findings are withdrawn from the regression results. While we used to expect a significant correlation between almost all-weather variables and the stock market, the results show that rain, humidity and wind speed anomalies exert no significant impact on all risk and return indicators regardless of the lockdown. This negligible effect is in line with previous research by Goetzmann et al. [36] which proves an insignificant influence of weather on trading activities. Additionally, Giang et al. [62] also indicates that there exists no link between precipitation and financial performance of manufacturing companies in Vietnam.

To conclude, our study has reaffirmed that stock market performance cannot be accounted for merely and exclusively by traditional finance theory. In order to justify financial market anomalies, other non-financial factors should also be taken into consideration. Our research has proved a negative correlation between meteorological variables and stock risks and returns, which can be attributed to irrational decisions induced by weather variation. This finding emphasizes the importance of mitigating the adverse impact of weather-related factors on the decision-making process.

Hence, it is advisable that investors adhere to a long-term investment strategy, create trading plans, and maintain patience and discipline, which helps them to avoid impulsive investment decisions driven by emotions, minimize biased behaviour and enhance rationality. Furthermore, our findings of the significant relationship between meteorological factors and stock market suggest the inclusion of weather anomaly and other economically neutral behavioural variables in asset-pricing models. From the perspective of competent authorities, it is necessary to provide support for financial literacy and promote information transparency, which would help investors make informed, data-driven investment decisions and reduce the potential impact of weather-related risks on their portfolios. Additionally, the rise in the Vietnamese stock market during the lockdown demonstrated the success of the government's COVID-19 response plan. Although the method adopted to respond may differ among nations according to their political features and socioeconomic characteristics, it is undeniable that a timely, deliberate, and flexible response from the beginning is needed. Based on our research, we advise the government to consider factors such as the weather that may affect investors' mood when regulating the market in order to foster investors' confidence and trust, assist them in making rational trade decisions, and ultimately increase the profitability of the stock market.

While our study makes notable contributions in providing a comprehensive analysis of the relationship between weather anomaly and the stock market, there still persist certain limitations that require consideration in order to guide future research endeavors. First, it is important to acknowledge that the aggregate data of the stock market utilized in our research may not provide a direct measurement of individuals' trading activities. Therefore, future studies are recommended to consider investigating the impacts of weather on trading and risk-taking activities, preferably utilizing individuals' trading data rather than relying solely on aggregated stock market data, provided it is available. In addition, our research has a limitation in the data set since the data is only collected from two biggest stock exchanges in Vietnam (namely HNX and HOSE). The two another developing exchanges in Vietnam, UPcom and OTC, are not involved in the model. Thus, in order to completely reflect the big picture of Vietnamese stock market, our suggestion for the future research is to spend greater time and effort diversifying the data.

## Authors’ contribution

Nguyen Thi Hoa Hong: Conceived and designed the experiments; Performed the experiments; Wrote the paper.

Pham Thi Mai Huong: Performed the experiments; Analyzed and interpreted the data; Wrote the paper.

Nguyen Yen Linh: Contributed reagents, materials, analysis tools or data; Wrote the paper.

## Data availability statement

The data would be made available upon request.

## Funding

No funding is available for this study.

## Declaration of competing interest

The authors declare that they have no known competing financial interests or personal relationships that could have appeared to influence the work reported in this paper.

## References

[1] Chen N.-F., Roll R., Ross S.A. (1986). Economic forces and the stock market. J. Bus..

[2] Ewing B.T. (2002). Macroeconomic news and the returns of financial companies. Manag. Decis. Econ..

[3] Wang S., Guo Z. (2020). A study on the co‐movement and influencing factors of stock markets between China and the other G20 members. Int. J. Finance Econ..

[4] Drakos K. (2010). Terrorism activity, investor sentiment, and stock returns. Rev. Financ. Econ..

[5] Hirshleifer D., Shumway T. (2003). Good day sunshine: stock returns and the weather. J. Finance.

[6] Cao M., Wei J. (2005). Stock market returns: a note on temperature anomaly. J. Bank. Finance.

[7] Floros C. (2008). Stock market returns and the temperature effect: new evidence from Europe. Appl. Financ. Econ. Lett..

[8] Goodfellow C., Schiereck D., Verrier T. (2010). Does screen trading weather the weather? A note on cloudy skies, liquidity, and computerized stock markets. Int. Rev. Financ. Anal..

[9] Lanfear M.G., Lioui A., Siebert M.G. (2019). Market anomalies and disaster risk: evidence from extreme weather events. J. Financ. Mark..

[10] Saunders E.M. (1993). Stock prices and Wall Street weather. Am. Econ. Rev..

[11] Eckstein D., Kunzel V., Schafer L. (2021).

[12] Tran T.H. (2018). The inflation-economic growth relationship: estimating the inflation threshold in Vietnam.

[13] Nasir M.A., Shahbaz M., Mai T.T., Shubita M. (2021). Development of Vietnamese stock market: influence of domestic macroeconomic environment and regional markets. Int. J. Finance Econ..

[14] World Health Organization (2023).

[15] (2019). The Vietnam National Administration of Tourism.

[16] World Health Organization (2020).

[17] Le S.M. (2020). Containing the coronavirus (COVID-19): lessons from Vietnam. https://Blogs.Worldbank.Org/Health/Containing-Coronavirus-Covid-19-Lessonsvietnam.

[18] Vietnam Ministry of Finance (2020).

[19] Hartley K., Bales S., Bali A.S. (2021). COVID-19 response in a unitary state: emerging lessons from Vietnam, Policy Design and Practice.

[20] Muhlack N., Soost C., Henrich C.J. (2022). Does weather still affect the stock market?. Schmalenbach J. Bus. Res..

[21] Szyszka A. (2007). From the efficient market hypothesis to behavioral finance: how investors' psychology changes the vision of financial markets. SSRN Electron. J..

[22] Hutchison T.W., Friedman M. (1954). Essays in positive economics. Econ. J..

[23] Shiller R.J., Fischer S., Friedman B.M. (1984).

[24] Tversky A., Kahneman D. (1979). Judgment under uncertainty: heuristics and biases. Science.

[25] Barber B.M., Odean T. (2008). All that glitters: the effect of attention and news on the buying behavior of individual and institutional investors. Rev. Financ. Stud..

[26] Shu H.-C., Chang J.-H. (2015). Investor sentiment and financial market volatility. J. Behav. Finance.

[27] Isen A.M., Patrick R. (1983). The effect of positive feelings on risk taking: when the chips are down. Organ. Behav. Hum. Perform..

[28] Karimi S., Liu Y.-L. (2020). The differential impact of “mood” on consumers' decisions, a case of mobile payment adoption. Comput. Hum. Behav..

[29] Di Muro F., Murray K.B. (2012). An arousal regulation explanation of mood effects on consumer choice. J. Consum. Res..

[30] Ramlee F., Sanborn A.N., Tang N.K.Y. (2017). What sways people's judgement of sleep quality? A quantitative choice-making study with good and poor sleepers. Sleep.

[31] Forgas J.P., George J.M. (2001). Affective influences on judgments and behavior in organizations: an information processing perspective. Organ. Behav. Hum. Decis. Process..

[32] Forgas J.P. (1995). Mood and judgment: the affect infusion model (AIM). Psychol. Bull..

[33] Szyszka A. (2011). Behavioral anatomy of the financial crisis. J. CENTRUM Cathedra.

[34] Bassi A., Colacito R., Fulghieri P., Sole Mio O. (2013). An experimental analysis of weather and risk attitudes in financial decisions. Rev. Financ. Stud..

[35] Kramer L.A., Weber J.M. (2012). This is your portfolio on winter. Soc. Psychol. Personal. Sci..

[36] Goetzmann W.N., Kim D., Kumar A., Wang Q. (2015). Weather-induced mood, institutional investors, and stock returns. Rev. Financ. Stud..

[37] Shafi K., Mohammadi A. (2020). Too gloomy to invest: weather-induced mood and crowdfunding. J. Corp. Finance.

[38] Tunyi A.A., Machokoto M. (2021). The impact of weather-induced moods on M& A performance. Econ. Lett..

[39] Chang T., Nieh C.-C., Yang M.J., Yang T.-Y. (2006). Are stock market returns related to the weather effects? Empirical evidence from Taiwan. Phys. Stat. Mech. Appl..

[40] Pardo A., Valor E. (2003). Spanish stock returns: where is the weather effect?. Eur. Financ. Manag..

[41] Shahzad F. (2019). Does weather influence investor behavior, stock returns, and volatility? Evidence from the Greater China region. Phys. Stat. Mech. Appl..

[42] Vo X.V. (2016). Finance in Vietnam - an overview. Afro-Asian J. Finance Account. (AAJFA).

[43] To M.T. (2022).

[44] Mazur M., Dang M., Vega M. (2021). COVID-19 and the march 2020 stock market crash. Evidence from S& P1500. Finance Res. Lett..

[45] Anh D.L.T., Gan C. (2021). The impact of the COVID-19 lockdown on stock market performance: evidence from Vietnam. J. Econ. Stud..

[46] Sadowski A., Galar Z., Walasek R., Zimon G., Engelseth P. (2021). Big data insight on global mobility during the Covid-19 pandemic lockdown. J Big Data.

[47] Johnson B. (2020).

[48] Ammar A., Brach M., Trabelsi K., Chtourou H., Boukhris O., Masmoudi L., Bouaziz B., Bentlage E., How D., Ahmed M., Muller P., Muller N., Aloui A., Hammouda O., Paineiras-Domingos L., Braakman-Jansen A., Wrede C., Bastoni S., Pernambuco C., Mataruna L., Taheri M., Irandoust K., Khacharem A., Bragazzi N., Chamari K., Glenn J., Bott N., Gargouri F., Chaari L., Batatia H., Ali G., Abdelkarim O., Jarraya M., El Abed K., Souissi N., Van Gemert-Pijnen L., Riemann B., Riemann L., Moalla W., Gomez-Raja J., Epstein M., Sanderman R., Schulz S., Jerg A., Al-Horani R., Mansi T., Jmail M., Barbosa F., Ferreira-Santos F., Simunic B., Pisot R., Gaggioli A., Bailey S., Steinacker J., Driss T., Hoekelmann A. (2020). Effects of COVID-19 home confinement on eating behaviour and physical activity: results of the ECLB-COVID19 international online survey. Nutrients.

[49] Deng T., Xu T., Lee Y.J. (2022). Policy responses to COVID-19 and stock market reactions - an international evidence. J. Econ. Bus..

[50] Nguyen T.T.H., Aya S. (2021). The effect of disease outbreaks on shrimp aquaculture and the role of cooperatives in the Mekong Delta. J. Int. Econ. Manag..

[51] Yahya F., Shaohua Z., Abbas U., Waqas M. (2021). COVID-19-induced returns, attention, sentiments and social isolation: evidence from dynamic panel model. Global Bus. Rev..

[52] Lee W.Y., Jiang C.X., Indro D.C. (2002). Stock market volatility, excess returns, and the role of investor sentiment. J. Bank. Finance.

[53] Rupande L., Muguto H.T., Muzindutsi P.-F. (2019). Investor sentiment and stock return volatility: evidence from the johannesburg stock exchange. Cogent Econ. Finan..

[54] Loriot B., Hutson E., Au Yong H.H. (2020). Equity-linked executive compensation, hedging and foreign exchange exposure: Australian evidence. Aust. J. Manag..

[55] Lakonishok J., Shapiro A.C. (1986). Systematic risk, total risk and size as determinants of stock market returns. J. Bank. Finance.

[56] Jiang X., Zaman M.A. (2010). Aggregate insider trading: contrarian beliefs or superior information?. J. Bank. Finance.

[57] Rahman M.L., Amin A., Al Mamun M.A. (2021). The COVID-19 outbreak and stock market reactions: evidence from Australia. Finance Res. Lett..

[58] Mirza H.H. (2012). Stock market returns and weather anomaly: evidence from an emerging economy. J. Econ. Behav. Stud..

[59] Loughran T., Schultz P. (2004). Weather, stock returns, and the impact of localized trading behavior. J. Financ. Quant. Anal..

[60] Symeonidis L., Daskalakis G., Markellos R.N. (2010). Does the weather affect stock market volatility?. Finance Res. Lett..

[61] Kathiravan C., Raja M., Chinnadorai K.M. (2018). Stock market returns and the weather effect in Sri Lanka. SMART Journal of Business Management Studies.

[62] Giang N.T.H., Hanh T.M., Hien P.T., Trinh N.T., Huyen N.T.K., Trang V.H. (2021). The impacts of climate change risks on financial performance: evidence from listed manufacturing firms in Vietnam.

[63] Schmittmann J.M., Pirschel J., Meyer S., Hackethal A. (2015). The impact of weather on German retail investors. Rev. Finance.

[64] Chuang Y.-W., Tsai W.-C., Weng P.-S. (2020). The impact of weather on order submissions and trading performance. Pac. Basin Finance J..

[65] Yuen K.S.L., Lee T.M.C. (2003). Could mood state affect risk-taking decisions?. J. Affect. Disord..

[66] Kliger D., Levy O. (2003). Mood-induced variation in risk preferences. J. Econ. Behav. Organ..

[67] Yan L., Li J., Liu M., Hu M., Xu Z., Xue K. (2021). Heating behavior using household air-conditioners during the COVID-19 lockdown in Wuhan: an exploratory and comparative study. Build. Environ..

[68] Bowen D.E., Schneider B. (2014). A service climate synthesis and future research agenda. J. Serv. Res..

[69] Howarth E., Hoffman M.S. (1984). A multidimensional approach to the relationship between mood and weather. Br. J. Psychol..

[70] Dallmann I. (2019). Weather variations and international trade. Environ. Resour. Econ..

